# Development of a KASP marker set for high-throughput genotyping in Japanese barley breeding programs with various end-use purposes

**DOI:** 10.1270/jsbbs.24052

**Published:** 2025-04-02

**Authors:** Hiroaki Shimizu, Goro Ishikawa, Hideyuki Aoki, Masaru Nakata, Junichi Tanaka

**Affiliations:** 1 Kyushu Okinawa Agricultural Research Center, National Agriculture and Food Research Organization (NARO), Izumi 496, Chikugo, Fukuoka 833-0041, Japan; 2 Institute of Crop Science, NARO, 2-1-2 Kannondai, Tsukuba, Ibaraki 305-8518, Japan; 3 Hokkaido Agricultural Research Center, Memuro Research Station, 9-4 Shinsei-Minami, Memuro, Kasai, Hokkaido 082-0081, Japan

**Keywords:** *Hordeum vulgare*, marker-assisted selection, automatic genotyping, breeding materials, barley type

## Abstract

In barley (*Hordeum vulgare* L.), many DNA markers have been developed for the selection of traits related to various end-use purposes of breeding. To perform rapid marker-assisted selection of many lines, we developed Kompetitive Allele-Specific PCR (KASP) markers, which can be used for effective automatic genotyping of single nucleotide polymorphisms (SNPs). The KASP primers were designed for 17 SNPs in 14 genes related to important traits. The target allele of all primers tried was identified on the basis of high FAM fluorescence in comparison with that of HEX. To evaluate the suitability of the developed markers in breeding programs, we used them to genotype 62 representative cultivars and lines. Then, using six of the developed markers, we comprehensively analyzed a total of 2,941 lines collected from eight breeding sites with a genotyping success rate of 95.1%–99.8% (mean, 98.6%). All six markers showed differences in allele percentages among breeding programs, and specific allele combinations were observed in all four barley types. Our data will be useful for predicting phenotype segregation and designing cross combinations. The developed KASP marker set can be used for high-throughput genotyping and should make breeding more efficient when combined with an accelerated generation technique.

## Introduction

In 2022, the global production of barley (*Hordeum vulgare* L.), the fourth largest crop in the world, was about 155 million tonnes (https://www.fao.org/faostat/en/#data/QC, accessed 2024/05/16). In Japan, barley is used for brewing (beer and whisky), food (pearled barley and *miso*), and barley tea. These end-use products are closely related to four barley types: two-row type is used mainly for pearled barley, six-row type is used for pearled barley as food and barley tea, malting type is used for beer and whisky, and naked type is used mainly for *miso*. Because its use depends on the region, different DNA markers at multiple breeding sites have been used.

The DNA markers proanthocyanidin-free (*ant28*) (Himi *et al.* 2012), amylose-free (*waxy*) (Aoki *et al.* 2017, Domon *et al.* 2002), high-lysine (*lys5*) (Patron *et al.* 2004), high-amylose (*amo1*) (Li *et al.* 2011), and low steely-grain rate (*fra*) (Aoki *et al.* 2024, Saito *et al.* 2018) for pearling, lipoxygenase-less (*lox-1*) (Oozeki *et al.* 2007), grain protein content (GPC) related to beer foam (*Protein Z4* and *Protein Z7*) (Iimure *et al.* 2011), and heat-stable β-amylase (*Bmy1*) (Paris *et al.* 2002) for malting have been developed and used for selection. In addition, Wang *et al.* (2015) reported that a single nucleotide polymorphism (SNP) in the coding region of the *NAM-1* gene has a significant effect on GPC. Since appropriate GPC is important in cultivars used for malting barley, a DNA marker that can detect this SNP could be useful for controlling GPC in breeding programs. DNA markers for the improvement of agronomic traits, such as resistance to barley yellow mosaic disease (*rym4* and *rym5*) (Nagamine *et al.* 2010, Stein *et al.* 2005) and seed dormancy, which inhibits field germination (*Qsd1* and *Qsd2*) (Nakamura *et al.* 2016, Sato *et al.* 2016), are used in almost all Japanese breeding programs. Since multiple SNPs have been reported in the coding regions of *Qsd1* and *Qsd2*, it is necessary to investigate these SNPs to accurately determine the alleles.

These DNA markers are genotyped mainly by gel electrophoresis. Cleaved amplified polymorphic sequence (CAPS) (Neff *et al.* 1998, Weining and Langridge 1991) and temperature switch PCR (Tabone *et al.* 2009) methods are frequently used to detect SNPs by gel electrophoresis. However, CAPS requires cost and time for restriction enzyme treatment, and primer design may be difficult for temperature switch PCR. Genotyping by next generation sequencing (NGS) can detect many SNPs and does not require gel electrophoresis (Bayer *et al.* 2017, Fan *et al.* 2006). Although the NGS approach can simultaneously survey many loci scattered across the genome, it is costly and time-consuming and is therefore unsuitable for investigating a specific gene in many samples in a short period. The competitive allele-specific PCR (called “Kompetitive Allele Specific PCR”, KASP) marker system is a method for SNP genotyping based on PCR with primers having bases corresponding to SNPs at the 3ʹ-end and detecting the fluorescence of the amplified product (Semagn *et al.* 2014) (https://www.biosearchtech.com/how-does-kasp-work, accessed 2022/04/14).

KASP markers can be used to rapidly analyze many samples because they can detect SNPs automatically without gel electrophoresis. They have been used for distinguishing genotypes in apple collections (Winfield *et al.* 2020) and stripe rust resistance genes of wheat (Wu *et al.* 2018). In barley, KASP markers have been developed for vernalization requirement, photoperiod reaction, and cleistogamy (Cockram *et al.* 2012), but these traits are not the main targets for marker-assisted selection in Japanese barley breeding.

In this study, we developed and validated a set of KASP markers that can be used to select for quality, disease resistance, and agronomic traits, which are the barley breeding targets in Japan. We used the markers to comprehensively estimate the genotypes of Japanese barley breeding materials and to examine the differences in allele percentages among breeding programs and in allele combinations among barley types. We also compared the conventional CAPS method and the KASP marker method in terms of the time, labor, and cost required for genotyping, and discuss the effectiveness of this marker set and its future use in barley breeding.

## Materials and Methods

### Plant materials

For the development of each KASP marker, one to three cultivars or breeding lines carrying the allele of interest were selected as positive controls. In addition, 62 leading Japanese cultivars, advanced breeding lines, and parental lines were used. For the *fra*, *Qsd1*, *Qsd2* and *rym5* genes, these 62 cultivars and lines have been genotyped using existing results of CAPS marker analysis and were used to evaluate the success and accuracy of the developed markers. For other genes, the reliability of developed makers was assessed using the positive controls listed in Supplemental Table 1.

To evaluate the suitability of the developed markers in segregating populations, we used 88 F_2_ plants for *ant28-484*, 192 F_5_ plants for *fra*, 96 F_2_ plants for *lox-1*, 96 F_2_ plants for *Qsd2-E7*, and 72 F_3_ plants for *waxSH97*. These are breeding populations in progress, and identification of heterozygotes is especially required for efficient selection of the target genes.

To confirm the versatility of the developed markers and to investigate differences in allele frequencies among breeding programs, we analyzed the genotypes of 2,941 lines collected from eight barley breeding sites in Japan which each have different breeding programs (Table 1).

### DNA extraction

Seeds of all materials, except for the *fra* population, were sown in a 9-cm polypod containing Nippi Engei Baido 1 soil (Nihon Hiryo Co., Ltd., Fujioka, Gunma, Japan) and grown at 22°C for 2–3 weeks. Young leaves (ca. 1 cm) were placed into a 2.0-mL tube. For the *fra* population, upper young leaves (ca. 1 cm) were collected in the field of the Institute of Crop Science, NARO (NICS). DNA was extracted as described in Ishikawa *et al.* (2022). In brief, a 6-mm bead and 400 μL of TPS buffer (100 mM Tris-HCl, pH 8.0, 10 mM EDTA, 1 M KCl) were added to each sample in a tube and the tube was shaken until the buffer became pale green. The sample was centrifuged for 15 minutes (min) at 7,200 × *g*, and 100 μL of the supernatant was transferred to a 1.5-mL tube and mixed with an equal volume of isopropanol. It was then centrifuged for 10 min at 16,000 × *g*, and the supernatant was carefully removed. The pellet was washed with 200 μL of 70% ethanol. The mixture was centrifuged for 3 min at 16,000 × *g*, the supernatant was carefully removed, and the pellet was air dried at room temperature. Sterile distilled water was added to dissolve the pellet. The DNA concentration of each sample was measured by Nanodrop (ND-ONEC-W, Thermo Fisher Scientific Inc., Waltham, MA, USA) and adjusted to 10 ng/μL with sterile distilled water, and the sample was stored at –20°C.

### KASP primer design

KASP primers were designed for 17 SNPs in 14 genes related to traits important for Japanese barley breeding. Details of the target genes are described in Supplemental Table 1. All target SNPs except for *amo1* are located in the coding regions of the genes and have been reported as important polymorphisms that distinguish alleles affecting their phenotypes. The *amo1* gene has not been characterized, but the target SNP is located in the vicinity of the causative gene, and it is known that the desired phenotype can be selected with this marker.

Using information obtained from the literatures listed in Supplemental Table 1, sequence data including SNPs were obtained from NCBI (https://www.ncbi.nlm.nih.gov/nuccore) or Ensembl Plants (https://plants.ensembl.org/index.html). The 3ʹ ends of the allele-specific primers were positioned on the SNP, and the primer length was adjusted in Net Primer software (http://www.premierbiosoft.com/netprimer/) so that the T_m_ values of the primers ranged from 60 to 65°C. The adaptor sequences of 5ʹ-gaaggtgaccaagttcatgct-3ʹ for one allele (primer labeled with FAM fluorescent dye) and 5ʹ-gaaggtcggagtcaacggatt-3ʹ for the other allele (primer labeled with HEX fluorescent dye) were added to the 5ʹ ends of the primers. Primers common to both alleles (COM) were designed in Primer3 software (https://primer3.ut.ee/) with settings of PCR product size 120–150 bp, T_m_ value 60–65°C, and GC rate close to those of the FAM/HEX primers. The designed primers were checked for self- and cross-dimerization and sequence specificity in Net Primer. When the ΔG of self- or cross-dimerization was below –10 kcal/mol, the primers were redesigned by adjusting the position of the COM primer or by using the complementary strand. Above, ΔG is the free energy of the primer calculated using the nearest neighbor method of Breslauer *et al.* (1986). In addition, the primer sequences were used as queries in BLAST searches against MorexV3_pseudomolecules_assembly in Ensembl Plants. Primers were redesigned if they matched sequences other than those near the 3ʹ end or if they had fewer than 3 base differences from the MorexV3 genome sequences other than the target genes. When none of the primers satisfied the above criteria, the specificity of the primer sequence was prioritized, and multiple primers were tested for their suitability in genotyping. Finally, the primer set that showed the best experimental results was selected and used as a marker for the target SNP (Table 2).

### KASP genotyping

KASP assay mix (100 μL) was prepared by mixing the FAM primer (12 μL; 100 μM), HEX primer (12 μL; 100 μM), COM primer (30 μL, 100 μM), and 46 μL of sterile distilled water. DNA samples (2 μL; 10 ng/μL) were dispensed into 384-well plates in duplicate. To each sample, a mixture of 2.5 μL of 2× Master Mix (LGC Limited, Teddington, UK), 0.17 μL of KASP assay mix, and 0.33 μL of sterile distilled water was added. A CFX384TM real-time PCR detector (Bio-Rad, Hercules, CA, USA) was used for PCR at 94°C for 15 min followed by 10 cycles of 94°C for 20 seconds (sec) and 61–55°C for 60 sec with a 0.6°C decrease per cycle and then 26 cycles of 94°C for 20 sec and 55°C for 60 sec, according to the KASP genotyping manual (https://biosearch-cdn.azureedge.net/assetsv6/KASP-genotyping-chemistry-User-guide.pdf). Fluorescence intensities were detected in a CFX384TM Optics Module real-time detector (Bio-Rad). Alleles were determined in CFX Maestro software (Bio-Rad). If the difference between FAM and HEX fluorescence intensities was small, three to six cycles at 94°C for 20 sec and 55°C for 60 sec were added and detection was repeated.

### Genotyping of 2,941 lines collected from eight breeding programs in Japan

Genotyping used 2,941 lines from eight barley breeding programs in Japan and six of the developed KASP markers—*Bmy1_SNP698*, *fra*, *lox-1*, *NAM-1_SNP544*, *Qsd1-E9*, and *waxSH97*. These genes are known to affect the end-use and were expected to vary in frequency among the breeding programs. The allele percentage for each marker was calculated for all lines from each breeding program. Samples with insufficient intensities of fluorescence or no amplification products were treated as missing data. The lines were classified into six-row, two-row, malting, and naked types on the basis of their end-use purposes and counted by the allele combinations of the six markers.

### Phenotype investivgation of the *waxSH97* population

Staining of *waxSH97* seeds with povidone-iodine gargle solution (7% Iodine Gargle, Meiji Seika Kaisha, Ltd., Tokyo, Japan) allows detection of the phenotype (Yanagisawa *et al.* 2006). The *waxSH97* population was used in a progeny test to confirm marker determination. The seeds of the F_2_ lines were genotyped as wild type (WT), homozygous for the allele of interest, or heterozygous. At least eight seeds of each F_2_ progeny line derived from ‘Kinumochi Nijo’ (*waxSH97*) × ‘YN004’ (WT) were used for each genotype.

## Results

### KASP marker development and validation for representative materials

**Fig. 1.**
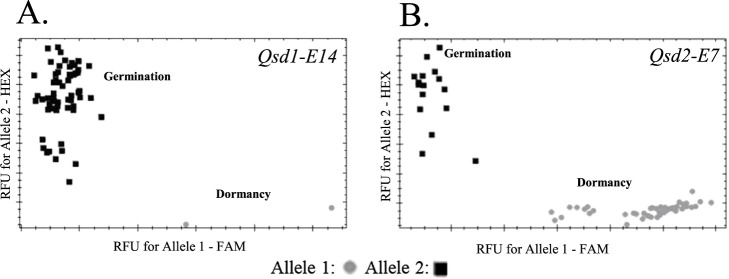
Genotype plots of 62 representative cultivars and lines of barley obtained with KASP markers (A) *Qsd1-E14* and (B) *Qsd2-E7*. RFU, relative fluorescence units. Genotype plots for other KASP markers are shown in Supplemental Fig. 1.

KASP primers designed for 17 SNPs in 14 genes are shown in Table 2. As expected, in all primer sets the FAM primer detected the allele of interest and the HEX primer detected the WT or unfavorable allele, making it possible to distinguish cultivars or lines with the alleles of interest (Fig. 1, Supplemental Fig. 1).

Using the KASP markers, we determined the genotypes of 62 cultivars and lines that have been important in barley breeding or are expected to be used as breeding materials. Eight markers—*lox-1*, *NAM-1_SNP544*, *ProteinZ4*, *ProteinZ7*, *Qsd1-E14*, *Qsd2-E7*, *rym5* and *waxSH97* determined the genotypes of all these materials (Supplemental Table 2). Other markers also showed high success rates in genotyping: 61 cultivars and lines (98.4%) by *Bmy1_SNP698*, *fra*, *lys5h(lys5g)*, *Qsd1-E11* and *rym4*; 59 (95.2%) by *amo1* and *ant28-484*; and 57 (92.0%) by *Qsd1-E9*. For those genotyped with existing CAPS markers, the genotype results were consistent for all developed KASP markers except *Qsd2-E7* (Supplemental Table 2). In *Qsd2-E7*, only three out of 60 cultivars or lines were discordant in genotype with the CAPS marker. Furthermore, all positive controls correctly showed FAM alleles (Supplemental Table 2). These results show that the developed markers can be used to characterize Japanese barley breeding materials.

### Application of developed markers to segregating populations

**Fig. 2.**
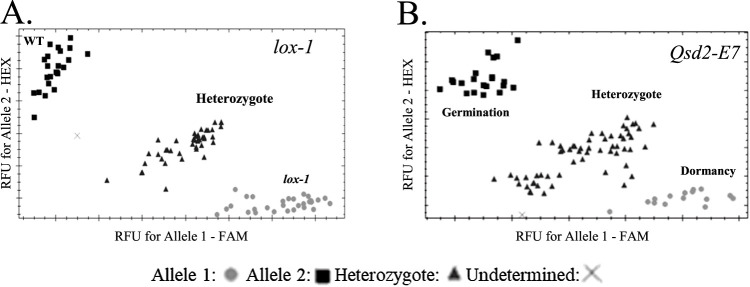
Genotype plots of the segregating population obtained with KASP markers (A) *lox-1* and (B) *Qsd2-E7*. RFU, relative fluorescence units. Genotype plots for *ant28-484*, *fra* and *waxSH97* populations are shown in Supplemental Fig. 1.

To evaluate whether the markers work in a co-dominant manner, we used the segregating populations *ant28-484*, *fra*, *lox-1*, *Qsd2-E7* and *waxSH97*. We determined the genotypes of 94.3% of plants in *ant28-484*, 92.3% in *fra*, 99.0% in *lox-1*, 99.0% in *Qsd2-E7* and 100% in *waxSH97* (Fig. 2, Supplemental Fig. 1). In the remaining samples, the intensity of fluorescence was insufficient to determine the genotype.

In the phenotype investigation of the *waxSH97* population, all seeds whose parental line was WT or *waxSH97* homozygous had the same phenotype as the parent. However, segregation of phenotypes was observed in seeds derived from heterozygous lines, confirming that the *waxSH97* marker identified heterozygous plants.

Furthermore, consistency between phenotypes and genotyping results was observed in the *ant28-484* population. However, this observation is not discussed in detail here, as not all populations have been subjected to the same level of study.

### Comprehensive KASP genotyping of Japanese barley breeding materials

Out of the 2,941 breeding materials, *Bmy1_SNP698* genotyped 2,796 (95.1%); *fra* genotyped 2,912 (99.0% of total); *lox-1* genotyped 2,931 (99.7%); *NAM-1_SNP544* genotyped 2,934 (99.8%); *Qsd1-E9* genotyped 2903 (98.7%) and *waxSH97* genotyped 2,914 (99.1%) (Table 3). The average success rate of the six markers was 98.6%. The number of samples with low fluorescence intensities or missing data was higher for *Bmy1_SNP698* than for the other markers.

Allele percentages of all six markers differed among breeding programs (Tables 1, 3). The percentage of the low steely-grain rate allele of *fra* was 7.6% overall, but it was relatively high at CARC and NICS. The WT allele of *waxSH97* was found in all lines at TARC and FARC, whereas the amylose-free allele was found in 25% of those at TAES and in 35.8% at WARC. The percentage of the foam and flavor stability allele of *lox-1* was generally low, except TAES (36.0%). The dormancy allele of *Qsd1-E9* was found in all lines at NAES, but only in 10% of lines at FARC.

The percentage of the high-protein allele of *NAM-1_SNP544* was much lower at TAES (1.4%) and FARC (4.3%) than the overall value (19%). The percentage of the allele for high-thermostability β-amylases of *Bmy1_SNP698* was highest at TAES (96.1%) and FARC (94.9%) and lowest at NICS (44.6%).

### Allele combinations among barley breeding materials

**Fig. 3.**
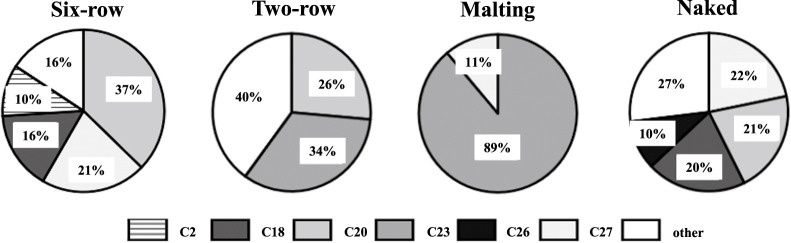
Allele combinations of the six developed KASP markers in the four barley types.

To investigate the relationship between uses and allele combinations, we used 2,611 lines (out of 2,941) with no missing or heterozygous data for any of the six markers. The lines were classified into six-row, two-row, malting, and naked types, and counted by allele combinations of the six markers (Fig. 3, Supplemental Table 3). Since barley type information was not available for 332 lines, 2,279 lines were finally classified into the four types. Of 64 theoretically possible combinations, 31 unique combinations were found (18 in six-row, 22 in two-row, 6 in malting, and 21 in naked) (C1 to C31). We also counted the combinations present in more than 10% of the lines and counted the lines with each combination. In the six-row type, four combinations (C2, C18, C20 and C27) were found in 904 of 1,073 lines. In naked, four combinations (C18, C20, C26 and C27) were found in 413 of 565 lines. In two-row, combinations C20 and C23 were found in 309 of 515 lines. In malting, C23 was found in 112 of 126 lines. Among >2000 lines from eight programs, most lines had one of the above allele combinations. Combinations that included the FAM allele of *NAM-1_SNP544* or the HEX allele of *Bmy1_SNP698* were present only in the six-row and naked types.

## Discussion

### Practicality and limitations of developed KASP marker set in barley breeding

KASP markers for 17 SNPs in 14 genes involved in important traits in Japanese barley breeding were developed and confirmed to discriminate alleles clearly in multiple lines (Fig. 1). Markers *ant28-484*, *fra*, *lox-1*, and *waxSH97* are available as co-dominant markers (Fig. 2) and will be effective in selecting early generations of cross populations in breeding programs.

Marker *Bmy1_SNP698* had the lowest percentage of allele determination (95.1% in 2941 lines), which could be explained by the effect of its paralogous gene, *Bmy2* (Vinje *et al.* 2011). Using the ‘Morex’ genome sequence of *Bmy1* (EU589328.1) and *Bmy2* (DQ889983.1), we compared the nucleotide sequences at the primer position of *Bmy1* with the corresponding position of *Bmy2*, and found that they differed only in three bases of the FAM primer (outside the SNP) and two bases of the COM primer (data not shown). This result indicates why the *Bmy1_SNP698* primer was less specific than the other primers. Although the call rate of the *Bmy1_SNP698* marker is lower than that of other markers, the high-throughput advantage of the marker is considered significant for breeding programs with multiple samples.

Since specificity is important in primer design (Ye *et al.* 2012), the presence of highly similar sequences in the genome must be carefully checked. In KASP marker design, it is necessary to design primers at the positions of SNPs, and thus the positions of the primers are restricted. Therefore, not all SNP markers can be converted into KASP markers, owing to the limitations of GC content, ΔG of self- or cross-dimerization, monotonously repeated sequence, primer specificity. It should also be noted that if the gene of interest has unexpected allele, it cannot be typed accurately. However, our approach to primer design is more flexible than other methods of detecting SNPs.

### Advantages of the developed KASP markers over conventional CAPS markers

The cost of SNP identification using a conventional CAPS marker varies owing to the wide range of restriction enzyme prices, but for a restriction enzyme available at a standard price the cost per 384 samples is approximately 18,700 JPY (Supplemental Table 4). Using a KASP marker, 384 samples can be genotyped for around 11,500 JPY. KASP markers require fewer steps and less time for genotyping than do CAPS markers because no gel electrophoresis is needed, and KASP markers are suitable for automated SNP genotyping owing to detection by fluorescence intensity. In breeding, many lines have to be genotyped with multiple markers in a short time, and the use of KASP markers could be a suitable method for marker-assisted selection (MAS).

### Differences in allele percentage among breeding programs

Analysis of 2,941 cultivars and breeding lines collected from eight barley breeding programs in Japan revealed the effect of end-use purposes at each breeding site, as described below (Tables 1, 3).

### SNPs selected in marker or phenotype selection: *fra*, *lox-1*, and *waxSH97*

The *fra* allele is useful to decrease the steely-grain rate, which has a negative effect on pearling time (Tonooka *et al.* 2010a). The percentage of the *fra* allele was highest at NICS (Pearled barley), with rich Andosols, where the steely-grain rate increases easily (Tonooka *et al.* 2010b). Therefore, lines with the *fra* mutant allele may have been preferentially introduced into lines at NICS.

The percentage of the *lox-1* (lipoxygenase-less) allele was highest at TAES. Lipoxygenase produces 2-nonenal, which diminishes beer flavor and foam stability (Kuroda *et al.* 2003). Beer made from the malt of *lox-1* lines has reduced contents of 2-nonenal (Hirota *et al.* 2006). TAES has been breeding beer barley, and our result may reflect the results of MAS of *lox-1* (Oozeki *et al.* 2007). On the other hand, the percentage of the *lox-1* allele was low (0.7%) at FARC where beer barley breeding is being performed. There are two possible reasons for this. One is that the marker was developed at TAES and may have been introduced into advanced breeding lines at an earlier stage. The other is that the allele may not have been prioritized at FARC. Further investigation is needed, but trait priorities, especially with regard to processing quality, may differ depending on manufacturer’s demand which may have influenced the different allele frequencies among breeding programs.

Barleys with no or low amylose content are used for pearling and are grown in many breeding programs. The mutant allele of *waxSH97*, which leads to the amylose-free phenotype, is derived from an artificial waxy mutant line of two-row barley (Domon *et al.* 2002). For this reason, the percentage of the *waxSH97* mutant allele was high at TAES, WARC, and KARC, where two-row barley is grown. Amylose-free cultivars in six-row barley areas (Table 1), such as ‘Haneumamochi’ (Aoki *et al.* 2017), bred at CARC, or ‘Kihadamochi’ (Tonooka *et al.* 2022), bred at NICS, have a *waxy* mutation different from *waxSH97*. Therefore, the *waxSH97* mutation is probably not widespread at CARC and NICS, which breed primarily six-row barley.

### SNPs that differ in suitable alleles depending on the intended purpose: *NAM-1_SNP544* and *Qsd1-E9*

The dormancy allele of *Qsd1-E9* enhances seed dormancy and inhibits field sprouting, but because of the germination delay, the malt quality is low. The percentage of the dormancy allele was low in the beer barley breeding programs (TAES and FARC) and high in the other programs.

*NAM-1_SNP544* affects protein content (Wang *et al.* 2015). High protein content decreases the quality of barley used for pearling and beer but increases that of barley used for tea (Mather *et al.* 1997, Matsuoka *et al.* 2010, Tonooka *et al.* 2010b). Six-row lines with the semi-dwarf gene (*uzu*) have been bred for barley tea at NICS. The relationship between *NAM-1_SNP544* and barley tea quality has not been reported, but *uzu* lines at NICS used for barley tea (*‘Kashima mugi’* and *‘Kashima goal’*) had the high-protein allele (Supplemental Table 2). These results suggest that the high-protein allele of *NAM-1_SNP544* may improve the quality of barley tea.

### Bmy1_SNP698 

*Bmy1_SNP698* is related to beer quality; the high-thermostability β-amylase allele was found in over 90% of lines in beer barley breeding programs (TAES and FARC) and in about 70% of lines in all programs except NICS. Since the NILs of ‘Shikoku Hadaka 84’, which lacks *Bmy1_SNP698* (Supplemental Table 2), are widely used as parental lines at NICS for introducing the *fra* allele, their progeny may reduce the overall *Bmy1_SNP698* allele percentage at NICS.

When pearl barley is cooked with rice, maltose is produced by barley β-amylase (Tsuyukubo and Kasai 2013). The high-thermostability β-amylase allele was found in at least about 70% of lines in all but one breeding programs; this β-amylase would increase the amount of maltose during mixed cooking with rice. In cooked rice, the combined content of maltose and maltotriose positively correlates with the aroma in taste tests (Maruyama *et al.* 1983). Therefore, the *Bmy1_SNP698* marker could be used to select lines of high quality for mixed cooking.

Our data showed an increase in the percentage of target alleles due to phenotype-based selection and genotypic selection during breeding. In the future, we may discover a novel marker–trait association by developing new markers. The information on genotypes obtained in this study would be useful for designing future crosses and selecting parents.

### Development of a versatile variety based on KASP genotyping

In Japan, barley is often processed for multiple uses. For example, ‘Shunrai’ is used for both pearling and barley tea. Low steely-grain rate is good for pearling, and high protein content is suitable for barley tea (Matsuoka *et al.* 2010). However, there is a positive correlation between the steely-grain rate and protein content (Tonooka *et al.* 2010b). Therefore, it isn’t easy to improve the suitability for both pearling and barley tea. The *fra* mutation decreases steely-grain rate and increases protein content in barley lines (Aoki *et al.* 2024, Saito *et al.* 2018). Therefore, we hypothesized that combining the FAM alleles of *fra* and *NAM-1_SNP544* would be beneficial for developing a versatile variety suitable for both barley tea and pearled barley. However, our genotyping found that only 1% of the lines analyzed had this combination (C1 and C3; Supplemental Table 3). To test this hypothesis, we intend to raise progeny using lines with C1 and C3 combinations as parents.

### Application of gene pyramiding by early generation selection

In Japanese barley breeding, MAS is generally performed in the F_4_ to F_6_ generations, when the frequency of homozygotes is increased. MAS using multiple markers in these generations would result in most individuals being discarded before subsequent phenotype-based selection. As desirable alleles accumulate, the number of loci subject to MAS increases, further reducing the efficiency of the selection. To avoid this problem, it is desirable to start MAS in earlier generations before the target alleles are genetically fixed. In addition, a combination of accelerating the generations using “speed breeding” technology (Watson *et al.* 2018) and appropriate MAS in the early a few generations can dramatically increase the desired allele frequencies. In this case, since a genotyping system with multiple co-dominant markers in a short time is required, the developed KASP markers will be ideal for this purpose. After accumulation of desirable alleles by MAS, phenotypic selection for complex traits such as yield should allow for more efficient breeding of promising lines. Furthermore, these promising lines can be used as parents for the next crosses to accelerate barley breeding. The combination of speed breeding with KASP markers described here, will accelerate the improvement of Japanese barley breeding.

## Author Contribution Statement

G.I. and J.T. designed the research; H.S. and H.A. performed the experiments; H.S. analyzed the data and wrote the original draft, M.N. provided guidance and advice on the Discussion section. All authors were involved in improving this manuscript. G.I. and J.T. are considered the corresponding authors since they shared the responsibility of supervising marker development and its breeding use, respectively.

## Supplementary Material

Supplemental Figures

Supplemental Tables

## Figures and Tables

**Table 1.** Barley breeding sites and programs in Japan, and number of lines analyzed

Abbreviation	Breeding site	Prefecture	Location	Main objective of breeding	Main barley type	No. of lines
TARC	Tohoku Agricultural Research Center, NARO	Iwate	40°N 141°E	Pearled barley	Six-row	236
CARC	Central Region Agricultural Research Center, NARO	Niigata	37°N 138°E	Pearled barley	Six-row	716
NAES	Nagano Prefectural Agricultural Experiment Station	Nagano	37°N 138°E	Pearled barley	Six-row	194
TAES	Tochigi Prefectural Agricultural Experiment Station	Tochigi	37°N 140°E	Barley beer	Two-row, Malting	288
NICS	Institute of Crop Science, NARO	Ibaraki	36°N 140°E	Pearled barley, barley tea	Six-row, Naked	548
WARC	Western Region Agricultural Research Center, NARO	Kagawa	34°N 134°E	Pearled barley	Six-row, Naked	354
KARC	Kyushu Okinawa Agricultural Research Center, NARO	Fukuoka	33°N 130°E	Pearled barley, *Shochu^a^*	Two-row	465
FARC	Fukuoka Agriculture and Forestry Research Center	Fukuoka	34°N 131°E	Barley beer	Two-row, Malting	140

*^a^* *Shochu* is a kind of liquor.

**Table 2.** KASP primer sequences and recommended number of PCR cycles

Gene/Target	SNP	Primer sequences*^a^*	PCR cycles*^b^*
*amo1*	G (High amylose)	FAM:gaaggtgaccaagttcatgctGCTGGCTCCGACTTTATTCG	32
	T (WT)	HEX:gaaggtcggagtcaacggattCGCTGGCTCCGACTTTATTCT	
		COM:GCTCAACATACGCAAAGCAGTG	
*ant28-484*	A (Proanthocyanidin-free)	FAM:gaaggtgaccaagttcatgctGCCTGAACAGAGGAGCGAGA	38
	G (WT)	HEX:gaaggtcggagtcaacggattGCCTGAACAGAGGAGCGAGG	
		COM:TGGAGACGATGGTGTGGGTAAC	
*Bmy1_SNP698*	C (High-thermostability β-amylases)	FAM:gaaggtgaccaagttcatgctGAGTGGGAATTTCCTAACGATGC	29
	T (Medium- or low-thermostability β-amylases)	HEX:gaaggtcggagtcaacggattCTGAGTGGGAATTTCCTAACGATGT	
		COM:CCGTGCTTGATCAGATTGTTGG	
*fra*	A (Low steely-grain rate)	FAM:gaaggtgaccaagttcatgctGGTAAATGGGCTCTCTGATGATTGA	32
	G (WT)	HEX:gaaggtcggagtcaacggattGTAAATGGGCTCTCTGATGATTGG	
		COM:ATACATTATTTCAAGCGCCATCTTG	
*lox-1*	T (Foam and flavour stability)	FAM:gaaggtgaccaagttcatgctCTACTCCATCAAGGCCATCACGT	32
	C (WT)	HEX:gaaggtcggagtcaacggattTACTCCATCAAGGCCATCACGC	
		COM:CTTGATGCCGCCCTCATAGAG	
*lys5h* (*lys5g*)	T (High lysine, shrunken endosperm)	FAM:gaaggtgaccaagttcatgctGCCTCAACCCTGTGCACCTATT	26
	C (WT)	HEX:gaaggtcggagtcaacggattGCCTCAACCCTGTGCACCTATC	
		COM:TGCAACACAAGTACACAACACAACG	
*NAM-1_SNP544*	C (High protein)	FAM:gaaggtgaccaagttcatgctGCGCGACCGCAAGTACC	29
	G (Not high protein)	HEX:gaaggtcggagtcaacggattGCGCGACCGCAAGTACG	
		COM:GCCGAGGCCAGGATAGGCTT	
*ProteinZ4*	A (Foam stability, pZ4-H)	FAM:gaaggtgaccaagttcatgctGTTCTACGAAATTCGGATACGAGGA	29
	G (WT, pZ4-L)	HEX:gaaggtcggagtcaacggattGTTCTACGAAATTCGGATACGAGGG	
		COM:CCATATGCACTTTGGTACTTTGGTAG	
*ProteinZ7*	C (Foam stability, pZ7-L and pZ7-L2)	FAM:gaaggtgaccaagttcatgctAGGCGGAGGTCGGTGGC	29
	T (WT, pZ7-H)	HEX:gaaggtcggagtcaacggattAGAGGCGGAGGTCGGTGGT	
		COM:CCTGTTAAATATCCACCCAACACCCA	
*Qsd1-E9*	G (High dormancy)	FAM:gaaggtgaccaagttcatgctGATTTTCGAAGTAAAGAGGTGCTTG	50
	C (Low dormancy)	HEX:gaaggtcggagtcaacggattGATTTTCGAAGTAAAGAGGTGCTTC	
		COM:ATTCACATTTATGGTATTGCAAGGTACA	
*Qsd1-E11*	G (High dormancy)	FAM:gaaggtgaccaagttcatgctAAGTTGCCAGATCACTTGGGTG	50
	A (Low dormancy)	HEX:gaaggtcggagtcaacggattGAAAGTTGCCAGATCACTTGGGTA	
		COM:CCATGGGTCTCTGCTCTAAGGC	
*Qsd1-E14*	A (High dormancy)	FAM:gaaggtgaccaagttcatgctCGCCAAGGCCGAGGACA	50
	G (Low dormancy)	HEX:gaaggtcggagtcaacggattCGCCAAGGCCGAGGACG	
		COM:AGCTTATGTAACACAGGCCCGAC	
*Qsd2-E6*	G (High dormancy)	FAM:gaaggtgaccaagttcatgctGGCAAAAATTACAGACTTTGGCG	50
	T (Low dormancy)	HEX:gaaggtcggagtcaacggattAGGCAAAAATTACAGACTTTGGCT	
		COM:GATTTGATGGCATTGCTACACTATTATAA	
*Qsd2-E7*	C (High dormancy)	FAM:gaaggtgaccaagttcatgctCATATATGTCACCTGAGAGAATTCGTAC	50
	A (Low dormancy)	HEX:gaaggtcggagtcaacggattCATATATGTCACCTGAGAGAATTCGTAA	
		COM:CTGGGCCTTCATTGACATTATATG	
*rym4*	T (Resistant to BaYMV)	FAM:gaaggtgaccaagttcatgctACAACCCGCAGGGCAAGTT	29
	C (Susceptible to BaYMV)	HEX:gaaggtcggagtcaacggatACAACCCGCAGGGCAAGTC	
		COM:AGAATCCAGTAAGAGAGGGGCT	
*rym5*	G (Resistant to BaYMV)	FAM:gaaggtgaccaagttcatgctGTGCCAATGGCGGTAAATGTAG	41
	C (Susceptible to BaYMV)	HEX:gaaggtcggagtcaacggattGTGCCAATGGCGGTAAATGTAC	
		COM:GTGAGAAGGGAATTAGGGTGGAAC	
*waxSH97*	A (Amylose free)	FAM:gaaggtgaccaagttcatgctGCAGAGAAGGCTGAAGCGCTA	44
	G (WT)	HEX:gaaggtcggagtcaacggattCAGAGAAGGCTGAAGCGCTG	
		COM:AGGTGCATGGTGATTGATGTCAG	

*^a^* Lowercase letters indicate tail sequences in primers. *^b^* Number of cycles at 94°C for 20 s and 55°C for 60 s.

**Table 3.** Allele percentages in Japanese barley breeding materials determined with the six KASP markers

Allele	*Bmy1_SNP698*		*fra*		*lox-1*		*NAM-1_SNP544*		*Qsd1-E9*		*waxSH97*	
	*C*		*A*		*T*		*C*		*G*		*A*	
Breeding site	%	Ratio*^a^*	%	Ratio*^a^*	%	Ratio*^a^*	%	Ratio*^a^*	%	Ratio*^a^*	%	Ratio*^a^*
TARC	73.2	169/231	7.0	16/230	0.4	1/235	33.9	80/236	93.6	218/233	0.0	0/234
CARC	69.0	457/662	13.3	95/712	2.8	20/714	18.1	129/714	90.7	643/709	2.2	16/715
NAES	80.8	147/182	2.6	5/193	2.6	5/194	20.6	40/194	100.0	193/193	1.6	3/193
TAES	96.1	269/280	0.3	1/286	36.0	103/286	1.4	4/287	36.9	103/279	25.0	71/284
NICS	44.6	227/509	16.0	86/539	0.9	5/548	24.1	132/548	86.6	472/545	6.4	35/547
WARC	70.0	238/340	0.6	2/351	0.0	0/351	34.1	120/352	94.3	331/351	35.8	121/338
KARC	67.6	307/454	3.2	15/463	1.5	7/463	9.7	45/463	77.3	350/453	18.6	86/463
FARC	94.9	131/138	0.0	0/138	0.7	1/140	4.3	6/140	10.0	14/140	0.0	0/140
Overall	69.6	1945/2796	7.6	220/2912	4.8	142/2931	19.0	556/2934	80.1	2324/2903	11.4	332/2914

*^a^* Number of FAM allele samples/Total number of fluorescent samples.
