# Supplementary material for: Development of a KASP marker set for high-throughput genotyping in Japanese barley breeding programs with various end-use purposes
Source: Breed Sci. 2025 Apr 2;75(2):129–38. doi: 10.1270/jsbbs.24052 (PMC12395202; doi:10.1270/jsbbs.24052)
Supplement: Supplementary file 1 — Supplemental Figures [file 75_129_s1.pdf]

Shimizu et al./Breeding Science
doi: 10.1270/jsbbs.24052

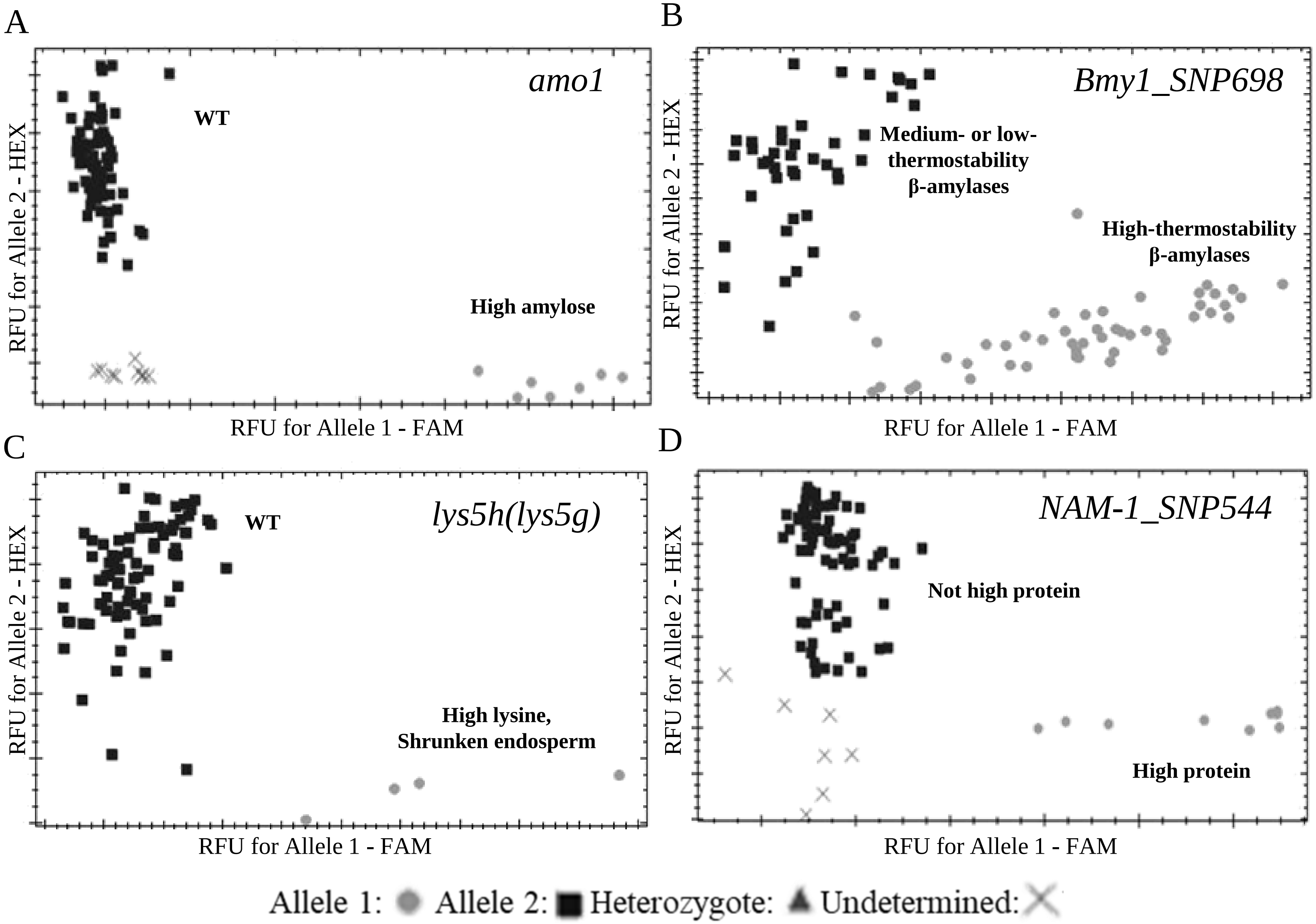

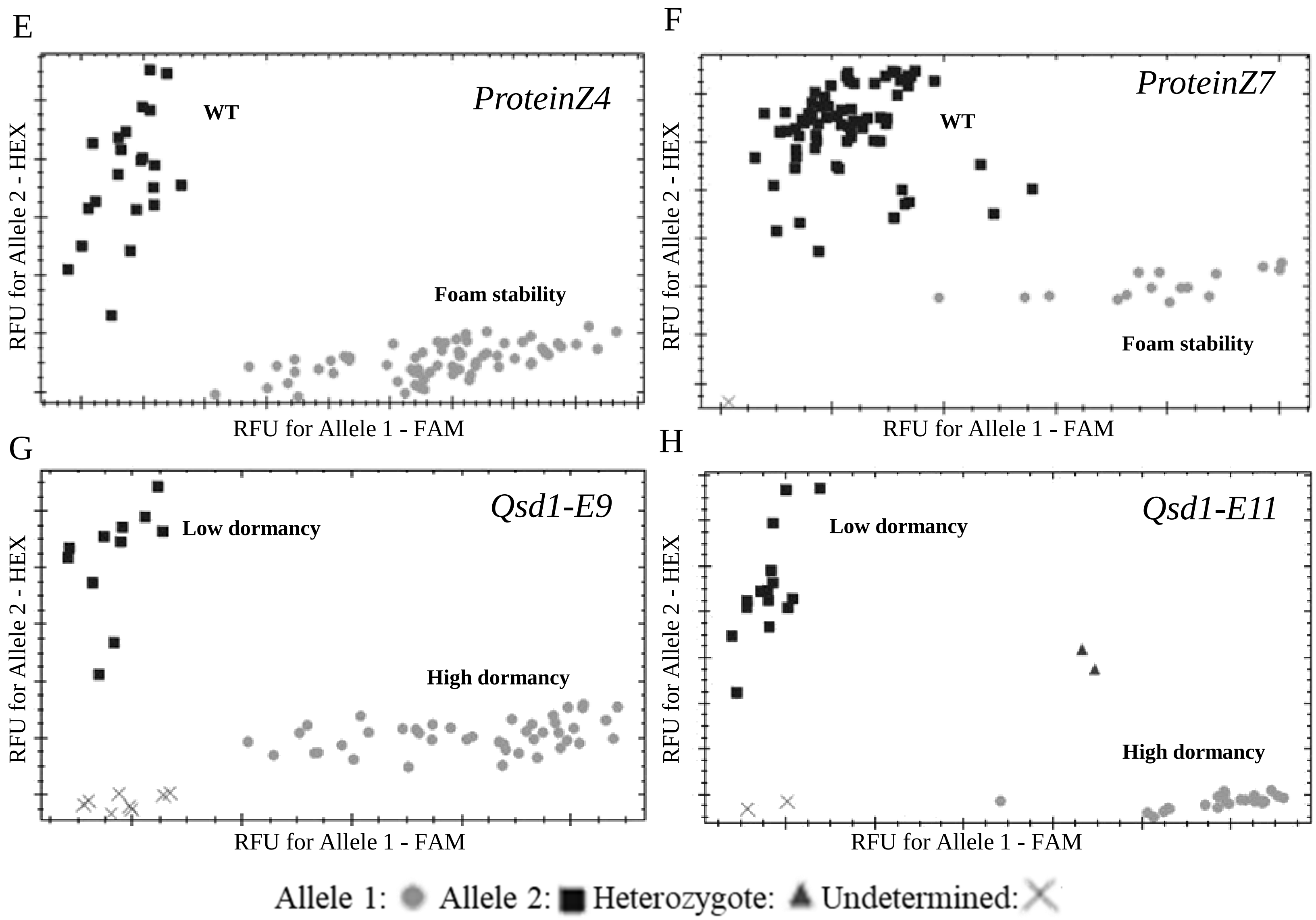

E
ProteinZ4
WT
Foam stability
RFU for Allele 2 - HEX
RFU for Allele 1 - FAM
F
ProteinZ7
WT
Foam stability
RFU for Allele 2 - HEX
RFU for Allele 1 - FAM
G
Qsd1-E9
Low dormancy
High dormancy
RFU for Allele 2 - HEX
RFU for Allele 1 - FAM
H
Qsd1-E11
Low dormancy
High dormancy
RFU for Allele 2 - HEX
RFU for Allele 1 - FAM
Allele 1: Allele 2: Heterozygote: Undetermined:

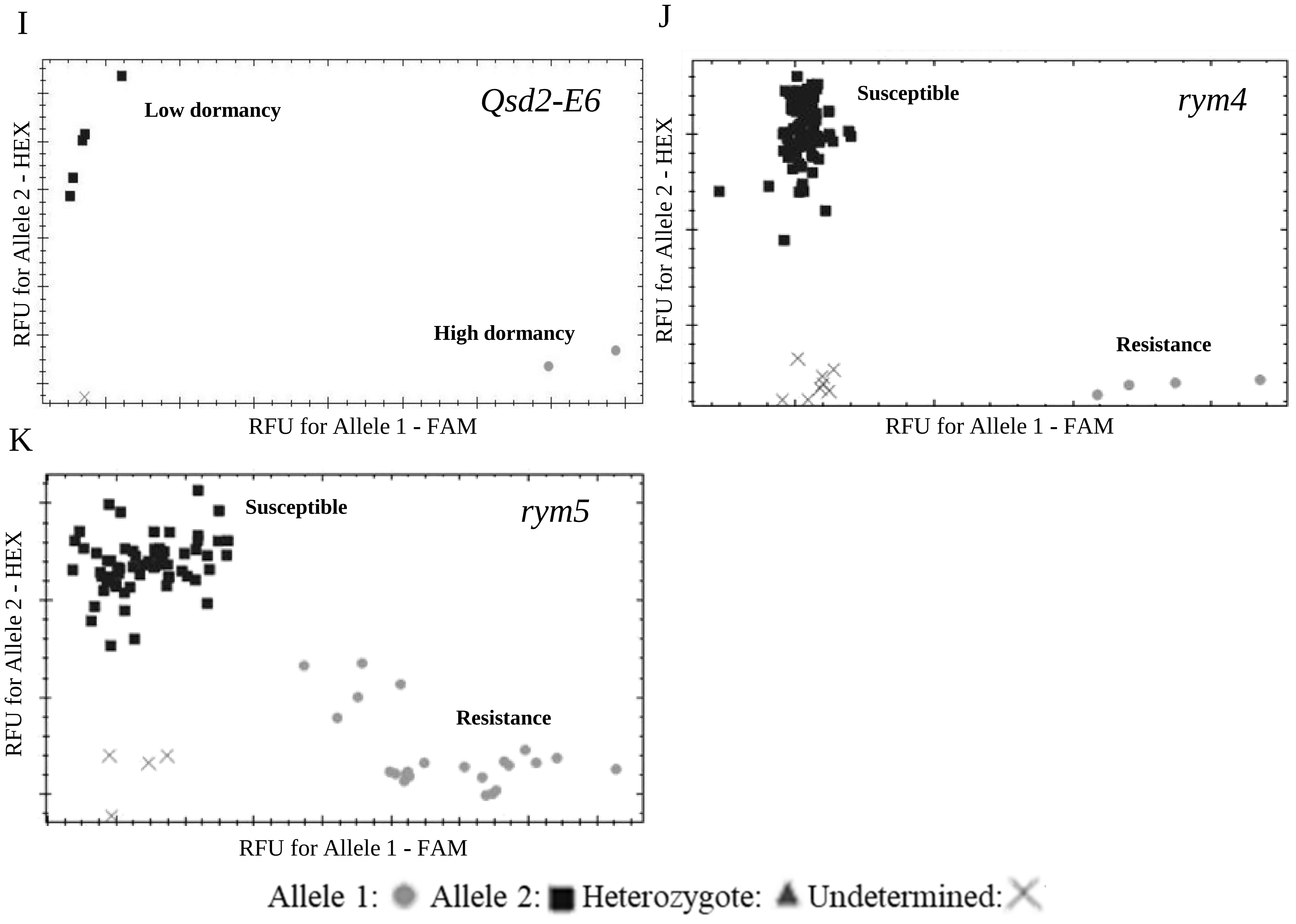

I
Qsd2-E6
Low dormancy
High dormancy
RFU for Allele 2 - HEX
RFU for Allele 1 - FAM
J
rym4
Susceptible
Resistance
RFU for Allele 2 - HEX
RFU for Allele 1 - FAM
K
rym5
Susceptible
Resistance
RFU for Allele 2 - HEX
RFU for Allele 1 - FAM
Allele 1: Allele 2: Heterozygote: Undetermined:

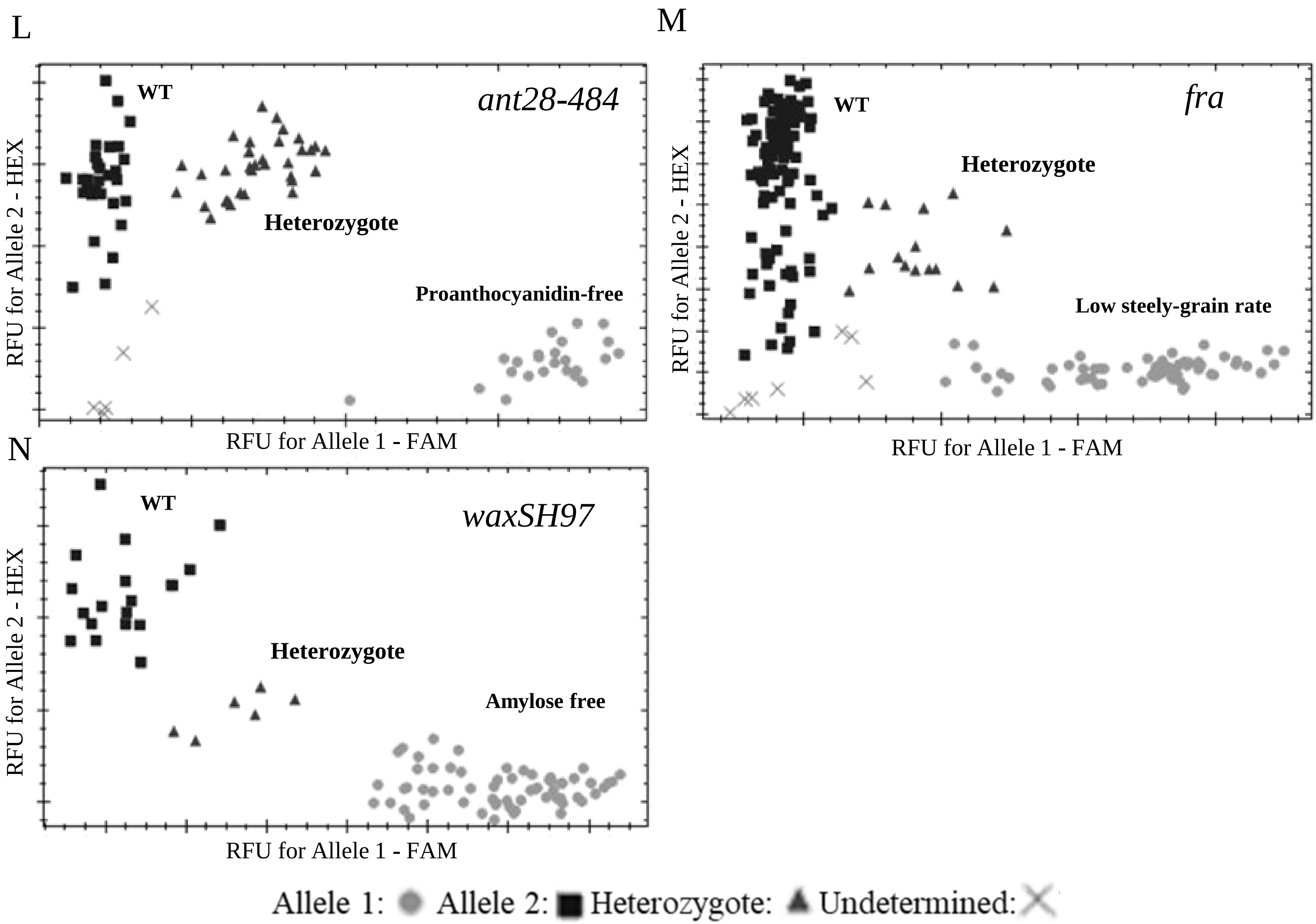
L
WT
ant28-484
Heterozygote
Proanthocyanidin-free
RFU for Allele 2 - HEX
RFU for Allele 1 - FAM
M
WT
fra
Heterozygote
Low steely-grain rate
RFU for Allele 2 - HEX
RFU for Allele 1 - FAM
N
WT
waxSH97
Heterozygote
Amylose free
RFU for Allele 2 - HEX
RFU for Allele 1 - FAM
Allele 1: Allele 2: Heterozygote: Undetermined:

**Supplemental Fig. 1.** Genotype plots obtained with developed KASP markers using barley cultivars and lines (A-K) and segregating populations (L-N). (A) *amo1*, (B) *Bmy1_SNP698*, (C) *lys5h(lys5g)*, (D) *NAM-1_SNP544*, (E) *ProteinZ4*, (F) *ProteinZ7*, (G) *Qsd1-E9*, (H) *Qsd1-E11*, (I) *Qsd2-E6*, (J) *rym4*, (K) *rym5*, (L) *ant28-484*, (M) *fra* and (N) *waxSH97*. RFU, relative fluorescence units.
