# Supplementary material for: Development of a KASP marker set for high-throughput genotyping in Japanese barley breeding programs with various end-use purposes
Source: Breed Sci. 2025 Apr 2;75(2):129–38. doi: 10.1270/jsbbs.24052 (PMC12395202; doi:10.1270/jsbbs.24052)
Supplement: Supplementary file 2 — Supplemental Tables [file 75_129_s2.pdf]

doi: 10.1270/jsbbs.24052

Supplemental Table 1. Target genes and SNP locations for KASP marker development.

Gene name	Gene ID[a]	Traits	Allele[b]	FAM control	Relation to trait	Target SNP location[c]		
						Chromosome	Position	Reference
amo1	HORVU.MOREX.r3.1HG0022500	High amylose	'GlacierAC38'	'Fukumi Fiber', 'Shikoku Hadaka 84 NIL(*amo1*)'	Tightly linked	1H	84434607	Li *et al.* 2011
ant28	HORVU.MOREX.r3.3HG0316260	Proanthocyanidin-free	*ant28-484*	'Fukumi Fiber', 'Kirarimochi'	Functional	3H	589151955	Himi *et al.* 2012
Bmy1	HORVU.MOREX.r3.4HG0417260	Thermostabilities for β-amylases	SNP698	'Haruna Nijo'	Functional	4H	606087055	Paris *et al.* 2002
fra	HORVU.MOREX.r3.4HG0334850	Low steely-grain rate	'Franubet'	'Shikoku Hadaka 84 NIL(*fra*)'	Functional	4H	9811428	Saito *et al.* 2018
lox-1	HORVU.MOREX.r3.4HG0335790	Beer foam and flavor stability	'Daikei LM1'	'Mochikinuka','New Sachiho Golden'	Functional	4H	12945076	Oozeki *et al.* 2007
lys5	HORVU.MOREX.r3.6HG0578050	High lysine	*lys5h(lys5g)*	'Shikoku Hadaka84 NIL(lys5h)', 'Riso29'	Functional	6H	186812424	Nakata *et al.* 2018, Patron *et al.* 2004
NAM-1	HORVU.MOREX.r3.6HG0556820	Protein content	SNP544	'Kashima Mugi'	Functional	6H	51376193	Wang *et al.* 2015
ProteinZ4	X51726.1	Beer foam stability	M3	'Satsuki Nijo'	Gene variation	4H	41831833	Iimure *et al.* 2011
ProteinZ7	HORVU.MOREX.r3.5HG0524850	Beer foam stability	Mkab1	'Barke'	Gene variation	5H	560134222	Iimure *et al.* 2011
Qsd1	HORVU.MOREX.r3.5HG0481320	Seed dormancy	*Qsd1-E9* [d]	'Kashima Mugi'	Functional	5H	435638651	Sato *et al.* 2016
Qsd1	HORVU.MOREX.r3.5HG0481320	Seed dormancy	*Qsd1-E11* [d]	'Mikamo Golden' / *H. vulgare* subsp. *spontaneum*	Gene variation	5H	435638233	Sato *et al.* 2016
Qsd1	HORVU.MOREX.r3.5HG0481320	Seed dormancy	*Qsd1-E14* [d]	'Kashima Mugi'	Gene variation	5H	435637064	Sato *et al.* 2016
Qsd2	HORVU.MOREX.r3.5HG0537390	Seed dormancy	*Qsd2-E6* [d]	'Kashima Mugi'	Gene variation	5H	585518949	Nakamura *et al.* 2016
Qsd2	HORVU.MOREX.r3.5HG0537390	Seed dormancy	*Qsd2-E7* [d]	'Kashima Mugi'	Functional	5H	585519163	Nakamura *et al.* 2016
rym4	HORVU.MOREX.r3.3HG0327270	Resistance to BaYMV	SNP170	'Franke', 'Iwate Mensury 2'	Functional	3H	612458352	Stein *et al.* 2005
rym5	HORVU.MOREX.r3.3HG0327270	Resistance to BaYMV	'Mokusekkou 3'	'Kirarimochi', 'Kusumochi Nijo'	Functional	3H	612460157	Nagamine *et al.* 2010
waxSH97	X07931.1	Amylose content	'Sikoku Hadaka97'	'Kirarimochi', 'Kusumochi Nijo', 'Shikoku Hadaka84 NIL(waxb)'	Functional	7H	17588220	Domon *et al.* 2002

[a] Gene ID obtained from Ensembl Plants or NCBI Blast.

[b] Name of allele(QTL) or derived cultivar or line.

[c] Target location of each SNP in MorexV3_pseudomolecules_assembly.

[d] *Qsd1-E9*, *E11*, *E14*, *Qsd2-E6* and *E7* indicate alleles containing non-synonymous SNP in the exon 9, exon 11 and exon 14 of *Qsd1* and exon 6 and exon 7 of *Qsd2*, respectively.

Supplemental Literature Cited

Nakata, M., T.M. Ikeda, Y. Ichinose, Y. Nogata, M. Seki, H. Aoki, T. Kato, K. Komae and T. Nagamine (2018) A novel allele of the ADP-glucose transporter gene in ‘Tanikei QM-1’ markedly increases health-promoting compounds in barley grains. Breed Res 20: 124-132 (in Japanese with English summary).

Supplemental Table 2. Genotypes of 17 target sites in 62 representative barley varieties and lines revealed with newly developed KASP markers.

Line	*amo1*	*ant28-484*	*Bmy1_SNP698*	*fra*	*fra* :CAPS[a]	*lox-1*	*lys5h(lys5g)*	*NAM-1_SNP544*	*ProtenZ4*	*ProteinZ7*	*Qsd1-E9*	*Qsd1-E9* :CAPS[a]	*Qsd1-E11*	*Qsd1-E14*	*Qsd2-E6*	*Qsd2-E7*	*Qsd2-E7* :CAPS[a]	*rym4*	*rym5*	*rym5* :CAPS[a]	*waxSH97*
Shikoku Line 1	HEX	HEX	HEX	HEX	WT	HEX	HEX	HEX	HEX	HEX	HEX	Germination	HEX	HEX	FAM	FAM	Dormancy	HEX	FAM	*rym5*	HEX
Shikoku Line 2	HEX	HEX	HEX	HEX	WT	HEX	HEX	HEX	FAM	HEX	HEX	Germination	HEX	HEX	FAM	FAM	Dormancy	HEX	HEX	WT	FAM
Shikoku Line 3	FAM	HEX	FAM	HEX	no data	HEX	HEX	HEX	FAM	FAM	FAM	Dormancy	HEX	HEX	FAM	FAM	Dormancy	HEX	HEX	WT	FAM
Shikoku Line 4	HEX	FAM	HEX	HEX	WT	HEX	HEX	FAM	FAM	FAM	FAM	Dormancy	HEX	HEX	FAM	FAM	Dormancy	HEX	HEX	WT	FAM
Shikoku Line 5	HEX	HEX	FAM	HEX	WT	HEX	HEX	FAM	FAM	FAM	FAM	Dormancy	HEX	HEX	FAM	FAM	Dormancy	HEX	HEX	WT	FAM
Shikoku Line 6	HEX	HEX	HEX	HEX	WT	HEX	HEX	HEX	FAM	HEX	FAM	Dormancy	HEX	HEX	FAM	FAM	Dormancy	HEX	FAM	*rym5*	FAM
Shikoku Line 7	HEX	HEX	FAM	HEX	WT	HEX	HEX	HEX	FAM	FAM	FAM	Dormancy	HEX	HEX	FAM	FAM	Dormancy	HEX	HEX	WT	HEX
Shikoku Line 8	HEX	HEX	FAM	HEX	WT	HEX	Undetermined	FAM	FAM	FAM	FAM	Dormancy	HEX	HEX	FAM	FAM	Germination	HEX	FAM	*rym5*	HEX
Tohoku Line 1	HEX	HEX	FAM	HEX	WT	HEX	HEX	HEX	FAM	HEX	FAM	Dormancy	HEX	HEX	FAM	FAM	Dormancy	HEX	HEX	WT	HEX
Tohoku Line 2	HEX	FAM	FAM	HEX	WT	HEX	HEX	HEX	FAM	HEX	FAM	Dormancy	HEX	HEX	FAM	FAM	Dormancy	HEX	HEX	WT	HEX
Tohoku Line 3	HEX	FAM	FAM	FAM	*fra*	HEX	HEX	HEX	FAM	HEX	FAM	Dormancy	HEX	HEX	FAM	HEX	Germination	HEX	HEX	WT	HEX
Tohoku Line 4	HEX	FAM	FAM	FAM	*fra*	HEX	HEX	HEX	FAM	HEX	FAM	Dormancy	HEX	HEX	FAM	FAM	Dormancy	HEX	HEX	WT	HEX
Tohoku Line 5	HEX	FAM	FAM	FAM	*fra*	HEX	HEX	HEX	FAM	HEX	FAM	Dormancy	HEX	HEX	FAM	FAM	Dormancy	FAM	HEX	WT	HEX
White Fiber	HEX	HEX	HEX	HEX	WT	HEX	HEX	HEX	FAM	HEX	FAM	Dormancy	HEX	HEX	FAM	FAM	Dormancy	HEX	HEX	WT	HEX
Tousan Line 1	HEX	HEX	FAM	HEX	WT	HEX	HEX	HEX	FAM	HEX	FAM	Dormancy	HEX	HEX	FAM	FAM	Dormancy	HEX	HEX	WT	HEX
Kihadamochi	HEX	HEX	HEX	HEX	WT	HEX	HEX	HEX	FAM	HEX	FAM	Dormancy	HEX	HEX	FAM	HEX	Dormancy	HEX	HEX	WT	HEX
Thukuba Line 1	HEX	HEX	FAM	HEX	WT	HEX	HEX	FAM	FAM	HEX	FAM	Dormancy	HEX	HEX	FAM	FAM	Dormancy	HEX	HEX	WT	HEX
Thukuba Line 2	HEX	HEX	FAM	HEX	WT	HEX	HEX	HEX	HEX	HEX	HEX	Germination	HEX	HEX	FAM	HEX	Germination	HEX	FAM	*rym5*	FAM
Thukuba Line 3	HEX	HEX	HEX	FAM	*fra*	HEX	HEX	HEX	FAM	HEX	FAM	Dormancy	HEX	HEX	FAM	HEX	Germination	HEX	HEX	WT	HEX
Thukuba Line 4	HEX	HEX	FAM	FAM	*fra*	HEX	HEX	HEX	FAM	HEX	FAM	Dormancy	HEX	HEX	FAM	FAM	Germination	HEX	HEX	WT	HEX
Thukuba Line 5	HEX	HEX	HEX	FAM	*fra*	HEX	HEX	HEX	FAM	HEX	FAM	Dormancy	HEX	HEX	FAM	FAM	Dormancy	HEX	HEX	WT	HEX
Kyushu Line 1	HEX	HEX	HEX	HEX	WT	HEX	HEX	HEX	HEX	HEX	FAM	Dormancy	HEX	HEX	FAM	HEX	Germination	HEX	HEX	WT	HEX
Kyushu Line 2	HEX	FAM	FAM	HEX	WT	HEX	HEX	HEX	FAM	HEX	FAM	Dormancy	HEX	HEX	FAM	HEX	Germination	HEX	FAM	*rym5*	FAM
Thochigi Line 1	HEX	FAM	FAM	HEX	WT	HEX	HEX	HEX	HEX	HEX	FAM	Dormancy	HEX	HEX	Undetermined	HEX	Germination	HEX	FAM	*rym5*	FAM
Ryohu	HEX	HEX	FAM	HEX	WT	HEX	HEX	HEX	HEX	HEX	Undetermined	Germination	HEX	HEX	FAM	HEX	Germination	HEX	HEX	WT	HEX
Fiber snow	HEX	HEX	FAM	HEX	WT	HEX	HEX	HEX	FAM	HEX	Undetermined	Dormancy	HEX	HEX	FAM	FAM	Dormancy	HEX	HEX	WT	HEX
Haneumamochi	HEX	HEX	FAM	HEX	WT	HEX	HEX	HEX	FAM	HEX	Undetermined	Dormancy	HEX	HEX	FAM	FAM	Dormancy	HEX	HEX	WT	HEX
Hokuriku Line 1	HEX	HEX	HEX	HEX	no data	HEX	HEX	HEX	FAM	HEX	Undetermined	Dormancy	HEX	HEX	FAM	FAM	no data	HEX	FAM	no data	HEX
Hokuriku Line 2	HEX	HEX	FAM	HEX	no data	HEX	HEX	HEX	FAM	HEX	Undetermined	Dormancy	HEX	HEX	FAM	FAM	Dormancy	HEX	HEX	WT	HEX
Hokuriku Line 3	HEX	Undetermined	FAM	HEX	WT	HEX	HEX	HEX	FAM	HEX	Undetermined	Dormancy	HEX	HEX	FAM	FAM	Dormancy	HEX	HEX	WT	HEX
Hokuriku Line 4	HEX	HEX	HEX	HEX	WT	HEX	HEX	FAM	FAM	HEX	Undetermined	Dormancy	HEX	HEX	FAM	FAM	Dormancy	HEX	HEX	WT	HEX
Hokuriku Line 5	HEX	HEX	FAM	FAM	*fra*	HEX	HEX	HEX	FAM	HEX	Undetermined	Dormancy	HEX	HEX	FAM	FAM	Dormancy	HEX	HEX	WT	HEX
Hokuriku Line 6	HEX	HEX	HEX	Undetermined	WT	HEX	FAM	HEX	HEX	HEX	FAM	Dormancy	HEX	HEX	FAM	HEX	Germination	HEX	HEX	WT	HEX
Hokuriku Line 7	HEX	HEX	HEX	HEX	WT	HEX	HEX	HEX	FAM	HEX	FAM	Dormancy	HEX	HEX	FAM	FAM	Dormancy	HEX	HEX	WT	FAM
Hokuriku Line 8	HEX	HEX	HEX	FAM	*fra*	HEX	HEX	HEX	FAM	HEX	FAM	Dormancy	HEX	HEX	FAM	FAM	Dormancy	HEX	HEX	WT	HEX
Hokuriku Line 9	HEX	HEX	HEX	HEX	WT	HEX	HEX	HEX	HEX	HEX	HEX	Germination	HEX	HEX	FAM	FAM	Dormancy	HEX	FAM	*rym5*	HEX
Hokuriku Line 10	HEX	HEX	FAM	HEX	WT	HEX	HEX	HEX	FAM	HEX	FAM	Dormancy	HEX	FAM	FAM	FAM	Dormancy	HEX	HEX	WT	HEX
Hokuriku Line 11	FAM	HEX	Undetermined	HEX	WT	HEX	HEX	HEX	FAM	FAM	FAM	Dormancy	HEX	HEX	FAM	FAM	Dormancy	HEX	HEX	WT	FAM
Hokuriku Line 12	FAM	HEX	FAM	HEX	WT	HEX	HEX	HEX	FAM	FAM	FAM	Dormancy	HEX	HEX	FAM	FAM	Dormancy	HEX	HEX	WT	FAM
Hokuriku Line 13	FAM	HEX	HEX	HEX	WT	HEX	HEX	HEX	FAM	FAM	FAM	Dormancy	HEX	HEX	FAM	FAM	Dormancy	HEX	FAM	*rym5*	FAM
Hokuriku Line 14	HEX	HEX	FAM	HEX	WT	HEX	HEX	HEX	HEX	HEX	FAM	Dormancy	HEX	HEX	FAM	FAM	Dormancy	HEX	HEX	WT	HEX
Hokuriku Line 15	HEX	HEX	HEX	HEX	WT	HEX	HEX	HEX	FAM	HEX	FAM	Dormancy	HEX	HEX	FAM	HEX	Germination	HEX	HEX	WT	HEX
Hokuriku Line 16	HEX	HEX	FAM	FAM	*fra*	HEX	HEX	HEX	FAM	HEX	FAM	Dormancy	HEX	HEX	FAM	FAM	Dormancy	HEX	FAM	*rym5*	HEX
Hokuriku Line 17	HEX	HEX	FAM	HEX	WT	HEX	HEX	HEX	HEX	HEX	FAM	Dormancy	HEX	HEX	FAM	FAM	Dormancy	HEX	HEX	WT	HEX
Hokuriku Line 18	HEX	HEX	HEX	HEX	WT	HEX	HEX	HEX	FAM	HEX	FAM	Dormancy	HEX	HEX	FAM	FAM	Dormancy	HEX	HEX	WT	HEX
Hokuriku Line 19	HEX	HEX	FAM	HEX	WT	HEX	HEX	HEX	FAM	FAM	HEX	Germination	HEX	HEX	FAM	HEX	Germination	HEX	FAM	*rym5*	HEX
Hokuriku Line 20	HEX	HEX	FAM	HEX	WT	HEX	HEX	HEX	FAM	FAM	HEX	Germination	HEX	HEX	FAM	HEX	Germination	HEX	FAM	*rym5*	HEX
Riso29	HEX	HEX	HEX	HEX	WT	HEX	FAM	HEX	FAM	FAM	HEX	Germination	HEX	HEX	FAM	HEX	Germination	HEX	HEX	WT	HEX
Chariot × Syunrai*2（B1F5)	HEX	HEX	HEX	HEX	WT	HEX	HEX	HEX	FAM	HEX	HEX	Germination	HEX	HEX	FAM	FAM	Dormancy	HEX	HEX	WT	HEX
Kusumochi Nijo	HEX	HEX	HEX	HEX	WT	HEX	HEX	HEX	FAM	HEX	HEX	Germination	HEX	HEX	FAM	FAM	Dormancy	HEX	FAM	*rym5*	FAM[b]
Shikoku Hadaka84	Undetermined	HEX	HEX	HEX	WT	HEX	HEX	HEX	FAM	HEX	FAM	Dormancy	HEX	HEX	FAM	FAM	Dormancy	Undetermined	HEX	WT	HEX
Shikoku Hadaka84 NIL（waxa）	HEX	HEX	HEX	HEX	WT	HEX	HEX	HEX	FAM	HEX	FAM	Dormancy	HEX	HEX	FAM	FAM	Dormancy	HEX	HEX	WT	HEX
Shikoku Hadaka84 NIL（waxb）	HEX	HEX	HEX	HEX	WT	HEX	HEX	HEX	FAM	HEX	FAM	Dormancy	HEX	HEX	FAM	FAM	Dormancy	HEX	HEX	WT	FAM[b]
Shikoku Hadaka84 NIL（fra）	HEX	HEX	HEX	FAM[b]	*fra*	HEX	HEX	HEX	FAM	HEX	FAM	Germination	HEX	HEX	FAM	FAM	Dormancy	HEX	HEX	WT	HEX
Shikoku Hadaka84 NIL（amo1）	FAM[b]	HEX	HEX	HEX	WT	HEX	HEX	HEX	FAM	HEX	FAM	Dormancy	HEX	HEX	Undetermined	FAM	Dormancy	HEX	HEX	WT	HEX
Shikoku Hadaka84 NIL（lys1）	HEX	HEX	HEX	HEX	WT	HEX	HEX	HEX	FAM	HEX	FAM	Dormancy	Undetermined	HEX	Undetermined	FAM	Dormancy	HEX	HEX	WT	HEX
Shikoku Hadaka84 NIL（lys5h）	HEX	HEX	HEX	HEX	WT	HEX	FAM[b]	HEX	FAM	HEX	FAM	Dormancy	HEX	HEX	Undetermined	HEX	Dormancy	HEX	HEX	WT	HEX
Ichibanboshi	HEX	HEX	FAM	HEX	WT	HEX	HEX	FAM	FAM	FAM	FAM	Dormancy	HEX	HEX	Undetermined	FAM	Dormancy	HEX	HEX	WT	HEX
Kashima mugi	HEX	HEX	FAM	HEX	WT	HEX	HEX	FAM[b]	FAM	HEX	FAM[b]	Dormancy	HEX	FAM[b]	FAM[b]	FAM[b]	Dormancy	HEX	HEX	WT	HEX
Kashima goal	HEX	HEX	HEX	HEX	WT	HEX	HEX	FAM	FAM	HEX	FAM	Dormancy	HEX	HEX	FAM	FAM	Dormancy	HEX	HEX	WT	HEX
Sachiho Golden	Undetermined	Undetermined	FAM	HEX	WT	HEX	HEX	HEX	HEX	HEX	HEX	Germination	HEX	HEX	FAM	HEX	Germination	HEX	HEX	WT	HEX
New Sachiho Golden	Undetermined	Undetermined	FAM	HEX	WT	FAM[b]	HEX	HEX	HEX	HEX	HEX	Germination	HEX	HEX	FAM	HEX	no data	HEX	HEX	WT	HEX
Fukumi Fiber[c]	FAM[b]	FAM[b]	no data	no data	no data	no data	no data	no data	no data	no data	no data	no data	no data	no data	no data	no data	no data	no data	no data	no data	FAM
Kirarimochi[c]	no data	FAM[b]	no data	HEX	WT	HEX	HEX	HEX	FAM	HEX	no data	no data	no data	no data	no data	no data	no data	HEX	FAM[b]	*rym5*	FAM[b]
Haruna Nijo[c]	no data	no data	FAM[b]	no data	no data	no data	no data	no data	no data	no data	no data	no data	no data	no data	no data	no data	no data	no data	no data	no data	no data
Mochikinuka[c]	no data	no data	no data	no data	no data	FAM[b]	no data	no data	no data	no data	no data	no data	no data	no data	no data	no data	no data	no data	no data	no data	no data
Satsuki Nijo[c]	no data	no data	no data	no data	no data	no data	no data	no data	FAM[b]	no data	no data	no data	no data	no data	no data	no data	no data	no data	no data	no data	no data
Barke[c]	no data	no data	no data	no data	no data	no data	no data	no data	no data	FAM[b]	no data	no data	no data	no data	no data	no data	no data	no data	no data	no data	no data
'Mikamo Golden' / *H. vulgare* subsp. *spontaneum*[c]	no data	no data	no data	no data	no data	no data	no data	no data	no data	no data	no data	no data	FAM[b]	no data	no data	no data	no data	no data	no data	no data	no data
Franke[c]	no data	no data	no data	no data	no data	no data	no data	no data	no data	no data	no data	no data	no data	no data	no data	no data	no data	FAM[b]	no data	no data	no data
Iwate Mensury 2[c]	no data	no data	no data	no data	no data	no data	no data	no data	no data	no data	no data	no data	no data	no data	no data	no data	no data	FAM[b]	no data	no data	no data

[a] Columns named CAPS indicate genotyping results with existing CAPS markers.

[b] Genotypes treated as positive controls are shown in red.

[c] Additional cultivars and lines used as positive controls of specific markers.

Supplemental Table 3. Allele combinations of the six KASP markers in 2611 Japanese barley breeding materials.

Combination	*Bmy1_ SNP698*	*fra*	*lox-1*	*NAM-1_SNP544*	*Qsd1-E9*	*wax SH97*	Number of lines	Rate (%)	Six-row	Rate in the type (%)	Two-row	Rate in the type (%)	Malting	Rate in the type (%)	Naked	Rate in the type (%)	Total
C1	FAM	FAM	HEX	FAM	FAM	HEX	22	0.84	21	1.96	0	0	0	0	0	0	21
C2	FAM	FAM	HEX	HEX	FAM	HEX	136	5.21	110	10.25	8	1.55	0	0	6	1.06	124
C3	HEX	FAM	HEX	FAM	FAM	HEX	4	0.15	4	0.37	0	0	0	0	0	0	4
C4	HEX	FAM	HEX	HEX	FAM	FAM	3	0.12	0	0	0	0	0	0	3	0.53	3
C5	HEX	FAM	HEX	HEX	FAM	HEX	37	1.42	29	2.7	0	0	0	0	2	0.35	31
C6	HEX	FAM	HEX	HEX	HEX	HEX	5	0.19	0	0	0	0	0	0	5	0.88	5
C7	FAM	HEX	FAM	FAM	FAM	FAM	2	0.08	0	0	2	0.39	0	0	0	0	2
C8	FAM	HEX	FAM	FAM	FAM	HEX	2	0.08	0	0	1	0.19	0	0	0	0	1
C9	FAM	HEX	FAM	HEX	FAM	FAM	39	1.49	0	0	24	4.66	0	0	0	0	24
C10	FAM	HEX	FAM	HEX	FAM	HEX	12	0.46	2	0.19	5	0.97	0	0	0	0	7
C11	FAM	HEX	FAM	HEX	HEX	FAM	18	0.69	0	0	11	2.14	1	0.79	0	0	12
C12	FAM	HEX	FAM	HEX	HEX	HEX	34	1.30	6	0.56	19	3.69	3	2.38	1	0.18	29
C13	HEX	HEX	FAM	HEX	FAM	FAM	1	0.04	0	0	0	0	0	0	0	0	0
C14	HEX	HEX	FAM	HEX	FAM	HEX	9	0.35	2	0.19	2	0.39	0	0	0	0	4
C15	HEX	HEX	FAM	HEX	HEX	FAM	5	0.19	0	0	0	0	0	0	5	0.88	5
C16	HEX	HEX	FAM	HEX	HEX	HEX	1	0.04	0	0	0	0	0	0	1	0.18	1
C17	FAM	HEX	HEX	FAM	FAM	FAM	17	0.65	0	0	1	0.19	0	0	13	2.3	14
C18	FAM	HEX	HEX	FAM	FAM	HEX	332	12.72	168	15.66	12	2.33	0	0	115	20.35	295
C19	FAM	HEX	HEX	HEX	FAM	FAM	82	3.14	7	0.65	12	2.33	0	0	50	8.85	69
C20	FAM	HEX	HEX	HEX	FAM	HEX	768	29.41	400	37.28	136	26.41	5	3.97	118	20.88	659
C21	FAM	HEX	HEX	FAM	HEX	HEX	4	0.15	1	0.09	2	0.39	0	0	0	0	3
C22	FAM	HEX	HEX	HEX	HEX	FAM	22	0.84	1	0.09	13	2.52	0	0	5	0.88	19
C23	FAM	HEX	HEX	HEX	HEX	HEX	333	12.75	6	0.56	173	33.59	112	88.89	13	2.3	304
C24	HEX	HEX	HEX	FAM	FAM	FAM	8	0.31	0	0	0	0	0	0	8	1.42	8
C25	HEX	HEX	HEX	FAM	FAM	HEX	119	4.56	85	7.92	5	0.97	0	0	10	1.77	100
C26	HEX	HEX	HEX	HEX	FAM	FAM	76	2.91	1	0.09	4	0.78	0	0	57	10.09	62
C27	HEX	HEX	HEX	HEX	FAM	HEX	422	16.16	226	21.06	28	5.44	1	0.79	123	21.77	378
C28	HEX	HEX	HEX	FAM	HEX	FAM	2	0.08	0	0	1	0.19	0	0	1	0.18	2
C29	HEX	HEX	HEX	FAM	HEX	HEX	2	0.08	0	0	1	0.19	0	0	1	0.18	2
C30	HEX	HEX	HEX	HEX	HEX	FAM	18	0.69	1	0.09	9	1.75	0	0	7	1.24	17
C31	HEX	HEX	HEX	HEX	HEX	HEX	76	2.91	3	0.28	46	8.93	4	3.17	21	3.72	74
Total							2611[a]	100	1073	100	515	100	126	100	565	100	2279[b]

[a] We used 2611 lines (out of 2941) with no missing or heterozygous data for any of the six markers.

[b] This number does not match the total because of the lack of type information for 332 lines.

Supplemental Table 4. Comparison of experimental chracteristics between conventional method (CAPS) and method adopted in this study (KASP).

Category		**CAPS**	**KASP**
Equipment		PCR machine	PCR machine and Plate reader/ qPCR machine
		Incubator	
		Electrophoresis device	
		Gel imager	
Reagents & consumables (cost per 384 samples, JPY)[a]		PCR polymerase (7000)	KASP master mix (10 000)
		PCR plates (1500)	PCR plates (1500)
		Restriction enzymes (8000)	
		Agarose (2200)	
		Electrophoresis buffer	
		Staining solution	
	Total cost of reagents and consumables (JPY)[a]	**18,700**	**11,500**
Experimental procedure (time per 384 samples)		Mixing DNA with reagent (1.0 h)	Mixing DNA with reagent (1.0 h)
		PCR (1.5–2.0 h)	PCR (1.5–2.0 h)
		Mixing PCR product with enzyme (1.0 h)	Detection (0.5 h)
		Restriction enzyme treatment (1.5 h)	
		Gel loading (0.5 h)	
		Gel electrophoresis (1.5 h)	
		Detection (1.0 h)	
	Total time	**6.5–8.5 h**	**3.0–3.5 h**

[a] Costs are current estimates.
